# Motivation and emotional distraction interact and affect executive functions

**DOI:** 10.1186/s40359-024-01695-9

**Published:** 2024-04-05

**Authors:** Michael K. Yeung, Jaden Cheuk-Hei Wan, Michelle Mei-Ka Chan, Sam Ho-Yu Cheung, Steven Chun-Yui Sze, Winnie Wing-Yi Siu

**Affiliations:** 1 Department of Psychology, The Education University of Hong Kong, Tai Po, Hong Kong China; 2 Department of Rehabilitation Sciences, The Hong Kong Polytechnic University, Hung Hom, Hong Kong, China

**Keywords:** Executive function, Emotion, Motivation, Shifting, Inhibition, Updating

## Abstract

Previous research on cool-hot executive function (EF) interactions has examined the effects of motivation and emotional distraction on cool EF separately, focusing on one EF component at a time. Although both incentives and emotional distractors have been shown to modulate attention, how they interact and affect cool EF processes is still unclear. Here, we used an experimental paradigm that manipulated updating, inhibition, and shifting demands to determine the interactions of motivation and emotional distraction in the context of cool EF. Forty-five young adults (16 males, 29 females) completed the go/no-go (inhibition), two-back (updating), and task-switching (shifting) tasks. Monetary incentives were implemented to manipulate motivation, and task-irrelevant threatening or neutral faces were presented before the target stimulus to manipulate emotional distraction. We found that incentives significantly improved no-go accuracy, two-back accuracy, and reaction time (RT) switch cost. While emotional distractors had no significant effects on overall task performance, they abolished the incentive effects on no-go accuracy and RT switch cost. Altogether, these findings suggest that motivation and emotional distraction interact in the context of cool EF. Specifically, transient emotional distraction disrupts the upregulation of control activated by incentives. The present investigation has advanced knowledge about the relationship between cool and hot EF and highlights the importance of considering motivation–emotion interactions for a fuller understanding of control.

## Highlights

We examined how motivation and emotional distraction affected executive functions.

Monetary incentives enhanced performance across executive function tasks.

Emotional distractors abolished the incentive effects on inhibition and shifting.

Motivation and emotion interact in the context of specific executive functions.

Transient emotional distraction disrupts the upregulation of control by incentives.

## Introduction

Executive function (EF) refers to a family of top-down cognitive processes important to most aspects of life [1]. Although EF is elusive and difficult to define, a distinction between cool and hot EF is now widely recognized [2]. Cool EF involves cognitive abilities activated in motivationally and emotionally neutral contexts. Converging empirical evidence has supported the unity and diversity of cool EF: cool EF consists of several interrelated yet separable components, which include a common EF component (that explains inhibition and other control processes), an updating-specific component, and a shifting-specific component [3, 4]. These processes are commonly assessed using computerized tasks, including the go/no-go, *n*-back, and task-switching tasks, respectively [5, 6]. In contrast, hot EF refers to cognitive control processes displayed in motivationally significant and/or emotionally salient circumstances. Although its structure has not yet been established, hot EF is believed to consist of the abilities to use reward information and to regulate one’s own emotions for achieving optimal performance [2, 5]. These processes are often assessed using decision-making tasks that involve motivation and emotion.

Because both cool and hot EF shape goal-directed behavior, there has been much interest in examining the interactions between them, or the effects of emotion and motivation on cool EF processes [7–11]. One approach to investigating such interactions is to use cool EF paradigms with incentivized and nonincentivized stimuli and/or emotional and nonemotional stimuli to determine the effects of motivation (e.g., reward manipulation) and/or emotional distraction (e.g., negative emotional faces) on task performance [5]. Accordingly, cool EF is indexed by task performance in the absence of emotion-laden or incentivized stimuli. On the other hand, hot EF is reflected by task performance in the presence of incentives or emotional distractors, which necessitate behavioral adjustment and emotional self-regulation to achieve optimal performance.

Motivation is known to have a substantial effect on cognitive task performance [9]. Incentives generally facilitate task performance by upregulating attentional resources devoted to the target stimuli and by promoting preparatory, or proactive, control [11]. In the context of EF, many studies have found that incentives improve task performance; however, the exact influence of incentives on behavior remains elusive. Specifically, some studies have found that monetary reward improves go reaction time (RT) on the go/no-go task [12, 13]. However, it might [12] or might not [13] significantly impair no-go accuracy. In addition, reward cues have been found to enhance *n*-back task accuracy independent of the memory load [14] or specifically when reward cues are presented consciously [15]. Furthermore, monetary reward has been shown to reduce the RT switch cost during the task-switching paradigm [16]. These and other previous studies on the effect of motivation on EF have examined each EF component separately. Therefore, whether reward manipulation influences the domain-general or domain-specific aspects of cool EF is still poorly understood.

Similarly, emotional stimuli have long been hypothesized to have an impact on cognitive task performance. Depending on stimulus and subject features (e.g., highly arousing stimuli and anxious individuals), task-irrelevant negative emotional stimuli may draw one’s attention away from processing the target stimuli, thereby interfering with task performance [9]. In the context of EF, however, the literature on the effect of negative emotional distractors on task performance is mixed. Some studies found that negative emotional stimuli led to slower RT and more false alarms on the go/no-go task [17–19]. Another study reported null effects using a similar task [12]. In addition, some studies reported poorer updating accuracy or RT in the presence of emotional (threat-related) distractors compared to neutral distractors [18, 20, 21]. In contrast, one study failed to observe such effects in younger adults [22]. Furthermore, two studies showed that negative emotional stimuli increased RT switch costs on the task-switching task [23, 24]. Moreover, the literature on the relationship between negative affect and cognitive control is also inconclusive. Some studies showed that negative emotional distraction or negative affect was associated with reduced reactive or proactive control [25–27]. In contrast, others found that reactive control, proactive control, or both blocked off the negative impact of emotional distractors [25, 28].

Although often both motivation and emotional distraction have been shown to modulate attention and affect cool EF task performance, whether and how they interact with each other remains poorly understood. Based on the literature, incentives may interact with emotional distractors in at least two possible ways. First, emotional distractors have been shown to reduce control [25–27]. Accordingly, they may impede the upregulation of attention or control activated by incentives, reducing the incentive effects on EF task performance. In contrast, incentives may promote (proactive) control that helps to attenuate the distracting influence of emotion [25, 28], lowering the impact of emotional distraction on EF task performance. To our knowledge, only a few studies have examined the effects of motivation and emotional distraction on attention. These studies found that reward-associated stimuli [29] or block-level reward manipulation [30] reduced the interfering effect of negative emotional distractors on RT during a visual search task. Thus, there is some evidence that incentives can inhibit attentional allocation to negative emotional stimuli, thereby lowering the detrimental impact of negative emotion on task performance. Nonetheless, the interactions of incentives and emotional distractors have rarely been examined in the context of EF tasks that require control.

To address this knowledge gap, we conducted a preliminary, systematic investigation to determine how both motivation (incentive manipulation) and emotional distraction (threat-related faces) interacted and influenced cool EF. A paradigm that held test stimuli and trial structure constant was used to manipulate updating, inhibition, and shifting tasks with or without the context of motivation and emotional distraction. We hypothesized that incentives would enhance cool EF task performance, whereas task-irrelevant emotional stimuli would impede performance. In addition, we expected that motivation and emotional distraction would interact with each other during EF tasks, due to one process blocking off the other process.

## Methods

### Participants

Forty-five young adults (16 males, 29 females) with a mean age of 20.7 years (*SD* = 0.7 years) were recruited from the Hong Kong Polytechnic University via campus advertisement. Inclusion criteria included: (1) age 18–39 years; (2) no history of any developmental, neurological, or psychiatric disorders; (3) normal or corrected-to-normal vision; and (4) right-handedness, assessed by the four-item version of the Edinburgh Handedness Inventory [31]. The sample size was determined based on a power analysis (G*Power 3.1.9.7) conducted using the effect sizes of the Incentives × Emotional Distractors interaction reported in previous studies [29, 30]. Based on a weighted average Cohen’s *f* of 0.49, a power of 0.80, an alpha level of 0.05, the estimated minimum sample size was 36. Assuming 20% unusable data, the sample size needed was 45. Written informed consent was collected from all participants prior to the study. This study was approved by the Human Subjects Ethics Sub-Committee at Hong Kong Polytechnic University (HSEARS20210119002-R1) and conducted in accordance with the Declaration of Helsinki. This study was not preregistered.

### Procedure

Eligible participants were invited to the university to take part in an hour-long experiment. After providing written informed consent, participants filled out background questionnaires and then performed an experimental paradigm with three tasks. These tasks assessed inhibition, updating, and shifting abilities in the presence and absence of motivationally or emotionally salient contexts. The test stimuli and trial structure were matched across tasks, thereby allowing comparison of the effects of emotional distraction and motivation among the three cool EF domains. Participants received HKD$50–100, depending on their task performance, as an incentive.

### The EF paradigm

**Fig. 1:**
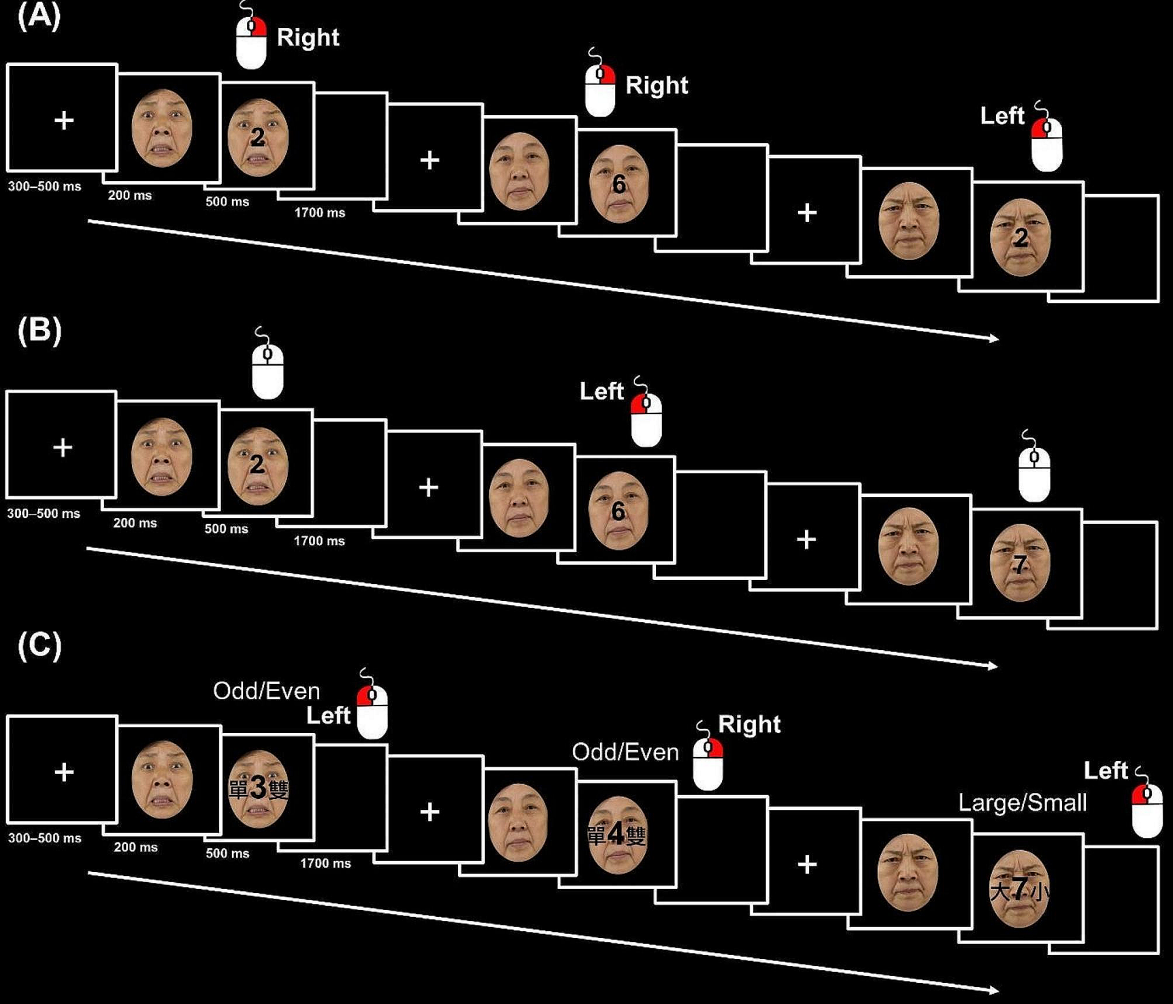
Flow of the task paradigms. Note: (A) The two-back (updating) task; (B) The go/no-go (inhibition) task; (C) The task-switching (shifting) task. The faces shown in this figure are taken from Yang et al. (2020) and modified and reused under the CC BY 4.0 license

Based on the EF literature [12, 16, 21], an experimental paradigm was utilized to manipulate updating (two-back), inhibition (go/no-go), and shifting (task-switching) demands (Fig. 1). Each task lasted approximately 20 min and consisted of four blocks of 96 trials per block: Two blocks were incentivized, and two were nonincentivized. During the incentivized block, participants were told that they would receive 50 cents for each correct and fast response. Reward feedback was presented at the end of each block, where the total sum of money earned in the last block was shown. To create the impression that only fast responses were rewarded, the total sum of money earned was calculated by rewarding correct responses only approximately two-thirds of the time. During the nonincentivized block, participants were told that they would receive no money for any response. The presentation order of the three tasks and the four blocks was randomized across participants. An instruction cue was presented before each block to inform participants of the upcoming incentive condition.

On each trial, a fixation cross was first presented at the center of a computer screen for 300–500 ms. Next, the photograph of either a neutral face or an angry or fearful face (i.e., threatening face) was presented with equiprobability at the center for 200 ms. After that, the face remained onscreen, and a single-digit, randomly drawn from 1 to 4 and 6–9, was superimposed on the face for 500 ms. According to the current task, participants were instructed to press the mouse button using their right index and middle fingers as fast and accurately as possible. Both the face and digit stimuli then disappeared, followed by a 1,700 ms interstimulus interval. The allowed response time limit was 2,000 ms. The intertrial interval varied from 2,600 to 2,800 ms.

The photographs were selected from the Tsinghua Facial Expression Database, based on the highest identification rate for the corresponding emotions [32]. Photographs of neutral and angry/fearful faces were taken from 48 Chinese adult actors, including 12 male younger adults, 12 female younger adults, 12 male older adults, and 12 female older adults.^1^ During each block, each actor appeared once for the nonemotion trials and once for the emotion trials. Anger and fear were chosen because they are both threat-related stimuli and are the most widely used negative emotions in emotion-cognition research [33, 34]. An oval mask was applied to each face to remove non-facial features.

In the present study, incentives were manipulated in blocks rather than in trials for various reasons. First, some evidence has suggested that both the trial-by-trial and block-by-block incentive manipulations affect task performance and physiological activity [12, 35]. Second, manipulating both incentive and emotion in trials would increase the stimulus load on each trial, which might lead to confusion and/or inadequate attention allocated to processing the stimuli. Third, due to the dependency of trials for some tasks (e.g., the two-back and task-switching tasks), trial-by-trial reward manipulation was not feasible. Moreover, only one task was used to assess each cool EF process for pragmatic reasons. Specifically, the three tasks took a total of one hour to complete, and adding additional tasks might lead to fatigue and habituation to the reward induction and emotional stimuli.

#### The two-back task

The two-back task was used to assess working memory updating (Fig. 1A). Upon seeing a digit, participants were required to judge whether the digit presented was the same as the one presented two trials ago. If it was the same (i.e., the target), they pressed the left key. If it was different (i.e., the nontarget), they pressed the right key. There were 26 target trials and 70 nontarget trials in each block. The dependent variables were the mean RT and accuracy on target and nontarget trials. Target and nontarget trials were separated because threat-related stimuli were found to affect task performance for specific trial types [18].

#### The Go/No-Go task

The go/no-go task was used to assess response inhibition (Fig. 1B). On each trial participants were asked to respond to a digit when shown, unless the digit was 2 or 7. There were 72 no-go trials and 24 go trials in each block. The dependent variables were the mean RT on go trials and accuracy on go and no-go trials.

#### The task-switching task

A cued task-switching task was adopted to assess attentional shifting (Fig. 1C). On each trial, when the digit stimulus was shown, a Chinese cue requiring magnitude judgment (“大 小”; large/small) or parity judgment (“單 雙”; odd/even) was presented at the center, surrounding the digit stimulus (e.g., “大 2 小”, “單 7 雙”). For the magnitude cue, participants pressed one button if the digit was larger than 5 and another button if the digit was smaller than 5. For the parity judgment cue, participants pressed one button if the digit was an odd number and another button if the digit was an even number. The stimulus-response mapping was counterbalanced across participants. In addition, a trial was considered as a repeat trial if the required judgment was the same as the one in the previous trial and as a switch trial if the required judgment was different from the one in the previous trial. Repeat and switch trials were equiprobable. The dependent variables were the mean RT and accuracy of repeat and switch trials.

### Data analysis

Accuracy and mean RT were analyzed for all three tasks. Mean RT was calculated based on correct trials only. Shapiro-Wilk tests showed significant deviation from normality for many accuracy and RT measures, with accuracy measures being negatively skewed and RT measures being positively skewed, *p*s < 0.05. Because square and log transformations are effective in reducing negative and positive skewness, respectively [36], these two transformation methods were applied to normalize the accuracy and RT measures, respectively, before parametric statistical analyses. Nevertheless, raw scores are presented in text and tables because they are easier to interpret.

To investigate the effects of emotional distraction and motivation on task performance, repeated-measures ANOVA with incentive (nonincentive, incentive), emotion (nonemotion, emotion), and condition as factors were conducted on the dependent variables. The condition was target and nontarget trials for the two-back task, go and no-go trials for the go/no-go task, and repeat and switch trials for the task-switching task. Significant interactions were followed by more repeated measures ANOVAs or paired *t*-tests with Bonferroni correction. Statistical analyses were performed using IBM SPSS Statistics for Windows, Version 26.0 (IBM Corp., Armonk, NY). The significance level was set at 0.05.

## Results

### Two-back task

**Fig. 2:**
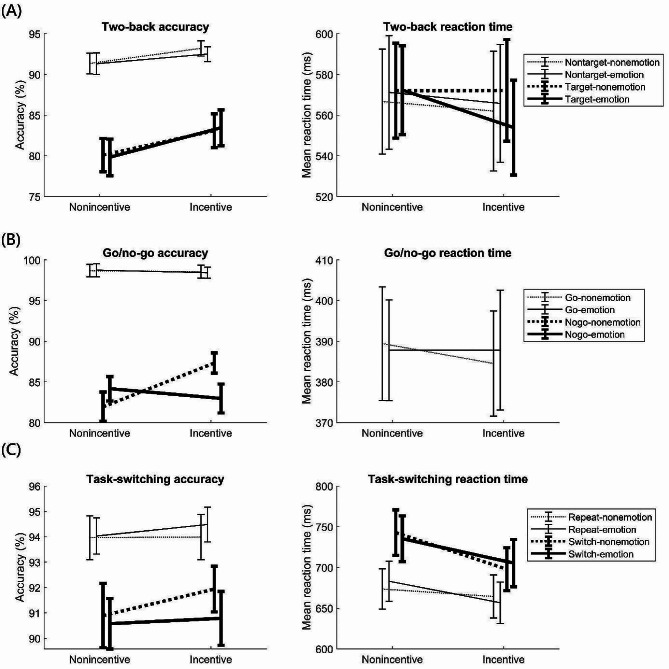
Accuracy and mean reaction time results. Note: (A) The two-back (updating) task; (B) The go/no-go (inhibition) task; (C) The task-switching (shifting) task. Error bars denote one standard error ± the means

The means and standard deviations of raw task measures are presented in Table 1; Fig. 2. First, the two-back task was analyzed (Table 1A). For log-transformed mean RT, the ANOVA showed no significant effects, *F*s < 2.24, *p*s > 0.14. For square-transformed accuracy, repeated measures ANOVA with incentive, emotion, and condition as factors yielded significant results for the main effects of incentive (incentivized > nonincentivized: *M* = 2.4%, *SD* = 7.0%), *F*(1, 44) = 6.80, *p* =.012, *η*^2^_p_ = 0.13, and condition (target > nontarget: *M* = 10.5%, *SD* = 10.4%), *F*(1, 44) = 52.34, *p* <.001, *η*^2^_p_ = 0.54, due to higher accuracy during the incentivized than nonincentivized block and during nontarget than target trials. No other effects were significant, *F*s < 2.52, *p*s > 0.12.

**Table 1:** Descriptive statistics of accuracy and reaction time (RT) measures

Measure	Factor			
	Nonincentive-nonemotion	Nonincentive-emotion	Incentive-nonemotion	Incentive-emotion
	Mean (*SD*)	Mean (*SD*)	Mean (*SD*)	Mean (*SD*)
(A) Two-back				
Nontarget accuracy (%)	91.3 (8.5)	91.3 (9.1)	93.2 (6.3)	92.5 (6.2)
Target accuracy (%)	80.1 (13.8)	79.8 (15.3)	83.1 (14.0)	83.5 (15.0)
Nontarget RT (ms)	567 (175)	571 (189)	562 (199)	566 (196)
Target RT (ms)	572 (158)	572 (148)	572 (169)	554 (158)
(B) Go/no-go				
Go accuracy (%)	98.7 (5.2)	98.7 (5.4)	98.6 (5.4)	98.4 (4.7)
No-go accuracy (%)	81.9 (12.3)	84.2 (10.2)	87.3 (8.5)	83.0 (12.1)
Go RT (ms)	389 (95)	388 (84)	385 (88)	388 (100)
(C) Task-switching				
Repeat accuracy (%)	94.0 (5.8)	94.0 (4.8)	94.0 (6.1)	94.5 (4.7)
Switch accuracy (%)	90.9 (8.6)	90.6 (6.7)	91.9 (6.1)	90.8 (7.2)
Repeat RT (ms)	673 (169)	683 (167)	664 (179)	657 (173)
Switch RT (ms)	743 (190)	735 (190)	698 (179)	705 (196)

### Go/no-go task

Next, the go/no-go task was analyzed (Table 1B). For log-transformed go RT, repeated measures ANOVA with reward and emotion as factors revealed no significant effects, *F*s < 0.68, *p*s > 0.41. For square-transformed accuracy, repeated measures ANOVA with incentive, emotion, and condition as factors yielded significant results for the main effect of condition (no-go > go: *M* = − 14.5%, *SD* = 9.7%), *F*(1, 44) = 144.71, *p* <.001, *η*^2^_p_ = 0.77, the incentive × condition interaction, *F*(1, 44) = 4.26, *p* =.046, *η*^2^_p_ = 0.088, and the incentive × emotion interaction, *F*(1, 44) = 7.56, *p* =.009, *η*^2^_p_ = 0.15. In addition, the incentive × emotion × condition interaction was significant, *F*(1, 44) = 5.36, *p* =.025, *η*^2^_p_ = 0.11. Thus, the incentive × emotion interaction was analyzed for the go and no-go trials separately. The *p*-value threshold was corrected to 0.025.

For go accuracy (i.e., hit rate), follow-up repeated measures ANOVA with incentive and emotion as factors revealed no significant results, *F*s < 0.68, *p*s > 0.41. For no-go accuracy (i.e., correct rejection rate), the ANOVA revealed a significant incentive × emotion interaction, *F*(1, 44) = 6.59, *p* =.014, *η*^2^_p_ = 0.13. Post-hoc paired *t*-tests with a corrected *p*-value threshold of 0.0125 revealed significantly higher accuracy during the incentivized than nonincentivized block for nonemotion trials (*M* = 5.4%, *SD* = 11.7%), *t*(44) = 3.04, *p* =.004, *d* = 0.45, but not emotion trials (*M* = − 1.2%, *SD* = 10.5%), *p* =.58. Therefore, negative emotional distractors abolished the incentive effect on no-go accuracy. There was no significant difference in no-go accuracy between emotion and nonemotion trials during the incentivized, *p* =.034, or nonincentivized block, *p* =.34.

### Task-switching task

The task-switching task was then analyzed (Table 1C). For log-transformed mean RT, repeated measures ANOVA with incentive, emotion, and condition as factors showed that the main effect of incentive (incentivized > nonincentivized: *M* = − 28 ms, *SD* = 62 ms), *F*(1, 44) = 9.44, *p* =.004, *η*^2^_p_ = 0.18, the main effect of condition (switch > repeat: *M* = 51 ms, *SD* = 34 ms), *F*(1, 44) = 162.62, *p* <.001, *η*^2^_p_ = 0.79, and the incentive × condition interaction, *F*(1, 44) = 6.61, *p* =.014, *η*^2^_p_ = 0.13, were significant. In addition, the incentive × emotion × condition interaction was also significant, *F*(1, 44) = 7.50, *p* =.009, *η*^2^_p_ = 0.15. Thus, the effects of incentive and condition were analyzed for the nonemotion and emotion trials separately, with *p*-value threshold corrected to 0.025.

For RT on nonemotion trials, repeated measures ANOVA with incentive and condition as factors yielded significant main effects of incentive (incentivized > nonincentivized: *M* = − 27 ms, *SD* = 38 ms), *F*(1, 44) = 7.51, *p* =.009, *η*^2^_p_ = 0.15, and condition (switch > repeat: *M* = 51 ms, *SD* = 32 ms), *F*(1, 44) = 160.02, *p* <.001, *η*^2^_p_ = 0.78. Importantly, there was a significant incentive × condition interaction, *F*(1, 44) = 14.39, *p* <.001, *η*^2^_p_ = 0.25, driven by a smaller RT switch cost during the incentivized than nonincentivized block (incentivized: *M* = 33 ms, *SD* = 43 ms; nonincentivized: *M* = 69 ms, *SD* = 52 ms). After correcting the *p*-value threshold to 0.0125, paired *t*-tests revealed significantly faster RT during the incentivized than nonincentivized block for switch trials (*M* = − 45 ms, *SD* = 73 ms), *t*(44) = 4.18, *p* <.001, *d* = 0.62, but not repeat trials (*M* = − 9 ms, *SD* = 79 ms), *t*(44) = 1.02, *p* =.32, *d* = 0.15.

Similarly, for RT on emotion trials, the ANOVA showed significant main effects of incentive (incentivized > nonincentivized: *M* = − 28 ms, *SD* = 63 ms), *F*(1, 44) = 9.49, *p* =.004, *η*^2^_p_ = 0.18, and condition (switch > repeat: *M* = 50 ms, *SD* = 44 ms), *F*(1, 44) = 83.60, *p* <.001, *η*^2^_p_ = 0.66. However, the incentive × condition interaction was not significant, *p* =.82. Therefore, during emotion trials, incentives improved repeat RT and switch RT to a similar extent but did not significantly reduce the RT switch cost (incentivized: *M* = 49 ms, *SD* = 54 ms; nonincentivized: *M* = 52 ms, *SD* = 53 ms).

To determine whether emotional distractors exerted an effect only in specific incentive contexts, the effects of emotional distraction and condition on RT were also analyzed for the incentivized and nonincentivized blocks separately. The *p*-value threshold was corrected to 0.025. Only the main effect of condition was significant for both the incentivized block (switch > repeat: *M* = 41 ms, *SD* = 42 ms) and nonincentivized block (switch > repeat: *M* = 61 ms, *SD* = 44 ms), *p*s < 0.001, due to slower RT during switch than repeat trials. No other effects were significant, *p*s > 0.027.

For square-transformed accuracy, repeated measures ANOVA with incentive, emotion, and condition as factors revealed a significant main effect of condition (target > nontarget: *M* = − 3%, *SD* = 3%), *F*(1, 44) = 35.84, *p* <.001, *η*^2^_p_ = 0.45, driven by significantly higher accuracy during repeat than switch trials. No other effects were significant, *F*s < 2.95, *p*s > 0.093.

## Discussion

In the present study, we examined simultaneously the effects and interactions of motivation (reward manipulation) and emotional distraction (threat-related faces) on cool EFs, including updating, inhibition, and shifting. A paradigm that implemented the two-back, go/no-go, and task-switching tasks while keeping the test stimuli and trial structure constant was adopted. We found that incentive induction significantly improved performance across the three EF tasks. Negative emotional stimuli had no significant main effects on any task performance; however, they modulated the effects of incentive manipulation on the go/no-go and task-switching tasks (but not the two-back task). These findings suggest that motivation and emotional distraction interact with each other and affect specific cool EF processes.

In the present study, incentive manipulation specifically improved no-go accuracy, two-back accuracy, and RT switch costs. In contrast, the induction of incentives had little influence on go RT, two-back RT, and repeat RT. Thus, incentives preferentially enhanced indicators of control while having little impact on visuomotor speed. These findings are in keeping with the putative role of motivation in increasing attentional allocation to anticipating and processing the target task stimuli and upregulating the neural substrates (e.g., the prefrontal cortex) of cognitive control [10].

Our findings are partially consistent with prior findings regarding how incentives influence cool EF task performance. For updating, the present two-back results are in keeping with previous findings of improved two-back accuracy during explicitly incentivized blocks [14, 15]. For inhibition, however, the present go/no-go results diverged from previous findings that incentives improved go RT while significantly lowering [12] or not affecting [13] no-go accuracy. Most previous studies manipulated incentives on a trial-by-trial basis, which induced the phasic effect of reward. In contrast, the present study manipulated incentives in blocks, which elicited the tonic effect of reward. Due to the distinction between phasic and tonic dopamine responses [37], this discrepancy could be due to the different way incentives were manipulated across studies. Nevertheless, for shifting, despite the difference in reward manipulation (i.e., in blocks vs. in trials), the present task-switching results are consistent with those reported in previous studies showing reduced RT switch cost following a cue that informed the availability vs. unavailability of reward in the next trial [16]. Thus, while the present study found that tonic reward manipulation enhanced task performance across cool EF processes, future work would benefit from determining whether the phasic and tonic modes of reward manipulation have varying effects on different cool EFs.

In this study, task-irrelevant negative facial stimuli had no significant main effect on any task performance. This adds to the controversial literature on the effects of negative emotional distractors on cool EF task performance, such as during the go/no-go [12, 17–19], *n*-back [20, 22], and task-switching tasks [23, 24]. The null results could be due to elimination of the disruptive effect of emotion by presenting task-irrelevant emotional stimuli at the same location as task-relevant stimuli. In addition, the effect of task-irrelevant emotional stimuli was found to be subject to attentional control instead of being entirely automatic [38]. The present participants, healthy college students, might be able to effectively allocate attention to task-relevant stimuli. Furthermore, although we used validated threat-related faces, the stimuli may not be arousing enough to compete with the task-relevant stimuli for perceptual processing [9]. Moreover, threatening and nonthreatening faces were intermixed in the present study, and some research has shown that both socially anxious and nonanxious individuals interpreted neutral faces as negative when they were under threat [39]. Thus, neutral expressions might influence attention like negative expressions. Factors that moderate the effects of emotional distraction on each EF process warrant further investigation.

Despite the lack of main effects, emotional distraction significantly influenced the incentive effects on go/no-go and task-switching performance. Specifically, when emotional distractors were present, incentives no longer benefited some performance indicators of control, including no-go accuracy and RT switch cost. This observation contrasts with previous findings that reward attenuated the effects of negative emotional distractors on visual search performance [25, 26]. Walsh et al. [30] manipulated reward between subjects and adopted a within-subject design to manipulate valence. Valence was varied in blocks rather than in trials, therefore emphasizing sustained emotional distraction. Yokoyama et al. [29] adopted a within-subject design and manipulated reward and valence on a trial-by-trial basis. A distractor was shown before the target stimulus that was associated with low or high reward. Therefore, participants were unknown of the incentive type upon seeing the distractor. In the present study, participants faced transient emotional distraction while anticipating reward throughout the incentivized block. Considering these study features, our results suggest that transient emotional distraction abolishes the enhancement of (proactive) control activated by incentives [11]. This interpretation aligns with a recent finding that transient emotional distraction disrupted the preparatory processes made available by temporally predictive cues [40].

According to the theory of motivational conflict [10], negative valence could interact with monetary incentives to create motivational conflict, requiring extra cognitive processing to evaluate the overall motivational state (i.e., appetitive or aversive). Thus, emotional stimuli may interfere with the current motivational state and abolish the effect of the selective improvement of reward induction on EF performances. It is, however, noteworthy that the interaction of incentives and emotional distractors was found only for the no-go and task-switching tasks but not the two-back task. The two-back task emphasized updating, which has been consistently demonstrated as a distinct component of EF [5]. Given the differentiated structure of EF and the employment of only one task per EF process in this study, more research is needed to clarify whether such interaction differs across EF components.

The amygdala circuits, mesolimbic dopaminergic pathways (e.g., ventral striatum and orbitofrontal cortex), and frontoparietal networks have been implicated in emotion processing, reward anticipation, and cognitive control, respectively [41–43]. Much research has suggested substantial overlaps and connections among these circuits [10, 44, 45]. Thus, the presently observed interactions of motivation and emotional distraction on influencing cool EF task performance could be due to interactions among these circuits. In addition, functional neuroimaging studies with both children and adults have found that updating, inhibition, and shifting engage overlapped but distinct regions in the brain, and these findings support the diversity and unity model of cool EF [42, 46]. Accordingly, the varying influences of emotional distractors and incentives on different EF tasks could be due to the moderation of different frontoparietal regions by the brain’s emotion and reward circuits. Future work would benefit from using functional magnetic resonance imaging to unravel the neural underpinning of our behavioral findings.

This study has several important implications. First, it has clarified the link between cool and hot EF, and thereby contributes to a fuller understanding of EF [2, 5]. In the present study, an experimental paradigm that manipulated updating, inhibition, and shifting demands was utilized to determine the effects of incentives and emotional distractors on various components of cool EF [4]. The results suggest that motivation interacts with emotional distraction in the context of specific cool EF processes. These findings will inspire further investigation on the interactions among motivation, emotion, and cognitive control, as well as the roles of task and subject characteristics and the neural basis of these interactions. In addition, we have developed an innovative task paradigm to facilitate a systematic assessment of motivation–emotion interactions in the context of EF. Alterations in reward and emotion processing (e.g., apathy) are present in many neuropsychiatric disorders, including schizophrenia [47] and Alzheimer’s disease [48, 49]. For example, avolition is a common characteristic of schizophrenia manifested by a decrease in reward-seeking behavior [50]. The current paradigm may be useful for capturing the impact of altered motivation and emotion on various core aspects of cool EF to improve diagnosis and treatment.

This study has some limitations. We used only one task to assess each cool EF because of time constraints and considerations about fatigue and habituation. There is consensus that EF consists of partially separable processes, which are most often probed by different tasks. Therefore, while the effects of motivation or emotion on EF have been studied using other tasks, such as the Stroop task [26, 51], whether the present findings regarding motivation–emotion interactions are generalizable to other task situations remains to be determined. Due to task impurity, future work would benefit from using multiple tasks per process to generalize the current findings to the latent construct level. In addition, we employed only verbal tasks. Some studies found that EF processes were modality independent [52], whereas others identified a mix of supramodal and modality-specific processes [53–55]. Hence, whether our findings are applicable to other modalities of the same tasks warrants further studies. Furthermore, this study studied only the behavior of healthy young adults. Future research applying this paradigm to other populations, using neurophysiological measurements, can help to further our knowledge about the impact of motivation and emotional distraction on goal-directed behaviors.

In summary, the present study examined the interactions of motivation and emotional distraction in the context of EF. We found that incentive induction enhanced behavioral performance, specifically indicators of control, across updating, inhibition, and shifting tasks. Emotional distractors had no significant main effect on the performance of EF tasks. However, it significantly moderated the impact of incentive induction on inhibition and shifting performance. Altogether, the findings suggest that transient emotional distractors interfere with the upregulation of control activated by incentives. This study provides evidence that specific EF processes are influenced by motivational and emotional processes and highlights the importance of considering motivation–emotion interactions for a fuller understanding of control.

## Data Availability

The data and materials are available from the corresponding author upon reasonable request.

## Footnotes

1 Y2M, Y15M, Y16M, Y24M, Y28M, Y30M, Y49M, Y55M, Y57M, Y58M, Y68M, Y75M, Y4F, Y5F, Y17F, Y23F, Y26F, Y31F, Y40F, Y48F, Y52F, Y65F, Y66F, Y71F, O08M, O15M, O17M, O42M, O50M, O55M, O58M, O63M, O64M, O67M, O69M, O71M, O19F, O22F, O23F-66, O24F-62, O26F, O29F, O40F, O43F, O47F, O48F, O49F, and O53F.
